# Anticancer effects of alpelisib on PIK3CA-mutated canine mammary tumor cell lines

**DOI:** 10.3389/fvets.2023.1279535

**Published:** 2023-11-15

**Authors:** Jiah Yeom, Yoonju Cho, Seoungyob Ahn, Soyoung Jeung

**Affiliations:** Research Institute, VIP Animal Medical Center, Seoul, Republic of Korea

**Keywords:** canine mammary tumor, PI3K, PIK3CA, alpelisib, canine cell line

## Abstract

Canine mammary tumors (CMTs) are commonly observed in old and unspayed female dogs. Recently, dogs have been increasingly spaying at a young age to prevent mammary tumors. These CMTs require extensive local excision and exhibit a high probability of metastasis to the regional lymph nodes and lungs during malignancy. However, the molecular and biological mechanisms underlying CMT development have not been fully elucidated, and research in this area is limited. Therefore, in this study, we established new CMT cell lines by isolating cells from tumor tissues and investigated phosphatidylinositol-4,5-bisphosphate 3-kinase catalytic subunit alpha (PIK3CA), a target for human breast cancer. PIK3CA mutations were observed at a similar loci as in the human PIK3CA gene in half of all canine samples. Furthermore, we investigated whether alpelisib, a PIK3CA inhibitor approved by the U.S. Food and Drug Administration for human breast cancer treatment, along with fulvestrant, is effective for CMT treatment. Alpelisib exerted stronger anticancer effects on cell lines with PIK3CA mutations than on the wild-type cell lines. In conclusion, we established new CMT cell lines with PIK3CA mutations and confirmed the efficacy of alpelisib for CMT treatment *in vitro*.

## Introduction

Canine mammary tumors (CMTs) are commonly observed in female dogs, and approximately 50% of all cases are malignant (1). Mastectomy, which involves tumor removal, is commonly used for CMT treatment; however, it is not completely effective as it only prevents possible dissemination of the tumor (2). Further *in vitro* research is necessary to develop alternative CMT treatment methods. Despite the increasing interest in CMTs, CMT-derived cell lines have only recently been developed for laboratory research (3–7), and are not as diverse as human cell lines. Many studies have analyzed the specific mutations in various genes, such as BRCA1, BRCA2, and phosphatidylinositol-4,5-bisphosphate 3-kinase catalytic subunit alpha (PIK3CA), using human breast cancer cell lines (8–11). Based on these studies, targeted therapies focusing on specific genes can be further developed. Although new canine cell lines have been established using patient-derived tissues (3–5), no canine CMT cell lines have been established to target specific mutations. Establishment of stable cell lines that highlight the characteristics of specific genes is necessary to develop effective targeted therapies for CMTs. Phosphoinositide 3-kinases (PI3Ks) are lipid kinases that regulate crucial cellular processes, such as cell proliferation, differentiation, and protein synthesis (12, 13). PI3Ks are divided into three classes: I, II, and III. Class I PI3Ks consist of heterodimers of the p85 regulatory and p110 catalytic subunits. They convert phosphatidylinositol (4,5)-bisphosphate (PI(4,5)P2) to the active signaling intermediate, phosphatidylinositol (3,4,5)-trisphosphate (PI(3,4,5)P3), which subsequently activates phosphoinositide-dependent kinase-1 (PDK1) and the protein kinase B (AKT) pathway. This activated signaling pathway plays an important role in various cell survival pathways. PI3K signaling pathway is involved in the development of human tumors (14, 15). PI3K/AKT signaling pathway positively regulates tumorigenesis, cancer cell metabolism, and drug resistance (15–18). Phosphatidylinositol-4,5-bisphosphate 3-kinase catalytic subunit alpha (PIK3CA), encoding the p110 subunit of class I PI3Ks, is highly activated and mutated in various human cancers, including the brain, liver, stomach, lung, and colon cancers (15, 18). In particular, breast cancer has a relatively low frequency of PIK3CA mutations compared to other cancers. However, recent studies have reported a high frequency (approximately 30%) of PIK3CA mutations (19, 20). Notably, mutations are frequently observed in the hotspots of exons 9 and 20, which encode the helical and catalytic domains, respectively (21, 22). The reported mutations, specifically PIK3CA E542K (c.1624G → A, p.Glu542Lys), E545K (c.1633G → A, p.Glu545Lys) in exon 9 and H1047R (c.3140A → G, p.His1047Arg), H1047L (c.3140A → T, p.His1047Leu) in exon 20, are missense mutations.

In addition to the importance of PIK3CA mutations in human breast cancer, various studies have shed light on their correlation with canine tumors (23–26). Tae-Min et al. reported that PIK3CA mutations are the most frequent somatic mutations in 43.1% of 183 CMT cases using whole exome sequencing (26). Interestingly, c.1637A > C and c.3140A > G mutations in CMT closely match those in human breast cancer (c. 1633G > A and c.3140 A > G) (27). Although other mutations are rarely detected in PIK3CA, a study reported the A3140G mutation in approximately 29% (18/62) of CMT specimens, which was higher than that observed in human breast cancer samples (14.3%) (24). Another study reported PIK3CA mutations at amino acid position 1,047 (H1047R, H1047L) in 45% (9/20) of hemangiosarcoma specimens of multiple breeds and 29.8% (14/47) of those of purebred dogs (25). Although various types of PI3K inhibitors have been developed, some drugs targeting multiple PI3K isoforms, such as copanlisib targeting the alpha and delta forms or taselisib inhibiting the alpha, delta, and gamma forms of PI3K, exhibit only moderate efficacy when combined with trastuzumab or fulvestrant for the treatment of patients with PIK3CA mutations (28). In contrast, alpelisib, a specific PI3K p110α inhibitor, combined with fulvestrant showed significant improvement and few side effects in patients with PIK3CA mutation in a clinical trial. Alpelisib was approved by the U.S. Food and Drug Administration (FDA) in 2019 and is currently used in combination with fulvestrant for the treatment of patients with PIK3CA-mutated breast cancer. Alpelisib is effective or human PIK3CA-mutated breast cancer treatment, and a high rate of PIK3CA mutations are observed in both human and canine mammary tumors. Therefore, whether alpelisib can be applied to canine patients requires further investigation. One study reported that alpelisib exerts potent anticancer effects on canine PIK3CA-mutated hemangiosarcoma-derived cells (23). PIK3CA mutations are commonly observed in two types of tumors, CMTs and hemangiosarcoma. Understanding the effects of alpelisib on CMT will facilitate its use for the treatment of canine diseases associated with PIK3CA mutations. Therefore, in this study, we aimed to identify PIK3CA mutations in CMT samples, establish novel CMT cell lines characterized with PIK3CA mutations, and examine the *in vitro* anticancer effects of alpelisib on these cell lines.

## Materials and methods

### Reagents

The normal canine Madin-Darby canine kidney (MDCK) cell line was purchased from the Korean Cell Line Bank (KCLB, Seoul, Korea). Dulbecco’s Modified Eagle’s Medium/nutrient mixture Ham’s F-12 (DMEM/F-12) was purchased from Corning (Corning, NY, United States). Fetal bovine serum (FBS) was purchased from Gibco (Thermo Fisher Scientific, Waltham, MA, United States). Trypsin-ethylenediaminetetraacetic acid (0.25%), phosphate-buffered saline (PBS), and penicillin/streptomycin were purchased from Welgene (Gyungsan, Korea). Cell viability assay kit and Mycoplasma polymerase chain reaction (PCR) detection kit were purchased from Dogen (Seoul, Korea) and Cellsafe (Gyeonggi, Korea), respectively. Alpelisib was purchased from Selleckchem (Munich, Germany).

### Primary tumor samples

A CMT cell line was established using a female canine clinically diagnosed with CMT. All surgically removed mammary tumor tissue samples were collected at the VIP Animal Medical Center (*n* = 5) and Haedeun Animal Medical Center (*n* = 7) after obtaining informed consent. After surgical removal, some tumor samples were fixed in formalin and embedded in paraffin (FFPE) for hematoxylin and eosin (H&E) staining. Veterinary pathologists conducted microscopic examinations to determine the tumor grade and evaluate cytological malignancy of the tumor cells. Other tumor sections were temporarily maintained in cold DMEM/F-12 for cell isolation.

### Cell isolation and establishment of cell line

VIP cell lines (VCLs) were generated via enzymatic dissociation. Tissue samples were washed with PBS, fragmented into small pieces, and dissociated via resuspension in DMEM/F-12 containing collagenase (Sigma, St. Louis, MO) for 4 h at 37°C in a humidified atmosphere containing 5% CO_2_. After centrifugation at 700 × g for 10 min, pelleted cells were carefully resuspended and cultured in DMEM/F-12 supplemented with 10% FBS and 1% penicillin/streptomycin. To eliminate the fibroblast contamination, trypsinization was performed as previously described (4). After reaching approximately 90% confluency, the cells were washed and incubated with cold 0.25% trypsin at room temperature for 2 min. The remaining cells were resuspended, sub-cultured approximately every 4–7 days, and cultured until the 30th passage. A total of 12 tumor samples were collected, and cell isolation was conducted. Among them, only 10 cells that grew successfully were used to establish cell lines. Cells between passages five and ten were used for subsequent experiments.

### DNA extraction and sequence analysis

Genomic DNA was extracted from cultured cell lines using the G-DEX™ IIc Genomic DNA Extraction kit (iNtRON Biotechnology Inc., Seoul, Korea), following the manufacturer’s instructions. Briefly, approximately 1 × 10^6^ cells were harvested and lysed using a lysis buffer containing RNase A. After incubation at 37°C for 30 min, protein precipitation buffer was added to the cell lysate, followed by brief centrifugation. The supernatant was transferred to a new tube and an equal volume of isopropanol (2-propanol) was added. After centrifugation, the pellet was washed with 70% ethanol and air-dried for 15 min. DNA rehydration buffer was added, and the extracted genomic DNA samples were submitted to Macrogen Inc. (Seoul, Korea) for Sanger sequencing. To examine the c.3140A > G (H1047R) mutation and c.1637A > C (Q546P) mutation, the following sequencing primer sets, originated from a previous publication (24), were used: 3140 primer set (Forward: 5’-CTG GAA TGC CAG AAC TAC AAT C-3′; Reverse: 5’-CTG TTC ATG GAT TGT GCA ATT CC-3′) and 1,637 primer set (Forward: 5’-TTC GCC ATT TTC TCT TTT TGT AGA-3′; Reverse: 5′-AGG TAT GGT AAA ACC TGC AAG ATA-3′). The amplified PCR products were purified and sequenced using an ABI PRISM 3730XL Analyzer. Variant analysis was conducted using the Variant Reporter Software (Version 2.1, Applied Biosystems), DNASTAR Lasergene SeqMan (Version 7.0), and Macrogen SNP analysis program (Version 1.0).

### Growth assay

To assess their growth characteristics, cells were seeded in a 6-well plate at a density of 5 × 10^4^ cells per well. After 24 h, each well was trypsinized, and the cells were microscopically counted for 6 days. Growth curves were plotted using GraphPad Prism (Version 9.5.0; Graph-Pad software Inc., San Diego, CA, United States), and the doubling time (DT) was calculated using an online tool.^1^

### Cell cytotoxicity assay

To evaluate the cytotoxic effect of alpelisib on CMT cell lines, the cells were seeded in a 96-well plate at a density of 1 × 10^5^ cells/mL. After overnight incubation at 37°C, the medium was replaced with serum-free DMEM/F-12, and cells were treated with alpelisib, which was dissolved in 1% final concentration of dimethyl sulfoxide (DMSO) at various concentrations (0, 0.01, 0.05, 0.1, 0.5, 1, 5, 10, 25, 50, and 100 μM). Cells were then incubated at 37°C for 24 h, 48 h, and 72 h. At the end of each treatment period, the medium was aspirated, and the EZ-cytox reagent (WST) was added to each well. After incubation at 37°C for 1 h, cell viability was measured at 450 nm using the LUMIstar Omega microplate reader (BMG labtech GmbH, Germany). Half-maximal inhibitory concentration (IC_50_) values were calculated using GraphPad Prism (Version 9.5.0; Graph-Pad software Inc.).

### Quantitative real-time polymerase chain reaction

Cells were seeded in a 12-well plate at a density of 1 × 10^5^ cells/mL. After overnight incubation at 37°C, the medium was replaced with serum-free DMEM/F-12, and cells were treated with alpelisib, which was dissolved in 0.1% final concentration of DMSO at various concentrations (0, 1, 5, and 10 μM). Then, the cells were incubated at 37°C for 24 h. Total RNA was extracted using the Trizol reagent (Thermo Scientific) and quantified using the NanoDrop™ One/One C Microvolume UV–Vis spectrophotometer (Thermo Scientific). After reverse-transcription to cDNA using the RevertAid First Strand cDNA Synthesis kit (Thermo Scientific), qRT-PCR was performed using the Quantstudio™ 1 Real-Time PCR System (Thermo Scientific) and Kapa SYBR Fast qPCR kit (Kapa Biosystems, Woburn, MA, United States). PCR was set up as follows: preheating to 95°C for 10 min, followed by 40 cycles at 95°C for 15 s, 60°C for 15 s, and 72°C for 30 s. β-actin was used as a reference gene. All primer sequences used in this study are listed in Table 1.

**Table 1:** Primer sequences used for qRT-PCR.

Gene	Forward (5′ → 3′)	Reverse (5′ → 3′)
*ACTB*	ACTGGGACGACATGGAGAAG	CATGGTTGGGGTGTTGAAGG
*SLC2A1*	GGTCCCTCTCTGTAGCCATC	CCAGTTTCGAGAAGCCCATG
*HK2*	TGCCTGGCTAACTTCATGGA	AAGTCCCCTCTCCTCTGGAT
*ESR1*	ATGTGTCCAGCTACCAACCA	CCAGAGGACCCCACTTCATT
*ERBB2*	GCATCCTGATCAAGCGAAGG	ACCTTCACCTTCCTCAGCTC
*PGR*	TGATTCAGATGCCAGCCAGA	TTATGCTGCCCTTCCATTGC

### Wound healing assay

To evaluate the migration capacity of CMT cell lines, the scratch assay was used. Cells were seeded in a 12-well plate at a density of 2.5 × 10^5^ cells/mL and allowed to grow to complete confluence. The center of resulting monolayers was then vertically scratched using a sterile pipet tip. After a brief washing with PBS, the medium was replaced with serum-free DMEM/F-12, and cells were treated with alpelisib, which was dissolved in 0.1% final concentration of DMSO at various concentrations (0, 1, 5, and 10 μM) and incubated at 37°C for 24 h and 48 h. Migration was examined by microscopy, and pictures were taken. Using ImageJ software (National Institutes of Health, Bethesda, MD, United States), the percentage of wound healing was calculated. Wound closure (%) was calculated based on the following equation: wound (%) = A (t0) − A (t) / A (t0) × 100.

### Mycoplasma detection

Mycoplasma DNA was detected using the BioMycoX^®^ Mycoplasma PCR Detection kit, following the manufacturer’s instructions. Briefly, the supernatant from cultured cells was collected, and PCR reaction was performed after mixing it with PCR premix and primer mix. PCR products were then separated using 1.5% agarose gel electrophoresis, and the presence of mycoplasma DNA band was confirmed under UV light using the Invitrogen™ iBright™ CL1500 Imaging System (Thermo Scientific).

### Statistical analysis

All experimental measurements were performed in triplicate, and data are expressed as the mean ± standard deviation (SD). Differences among groups were determined by one-way analysis of variance (ANOVA) with *post hoc* test (Turkey’s multiple comparison against control) to identify statistically significant differences using the GraphPad Prism software. Statistical significance was set as *p* value < 0.05.

## Results

### Histopathological examination and establishment of VCLs

**Figure 1:**
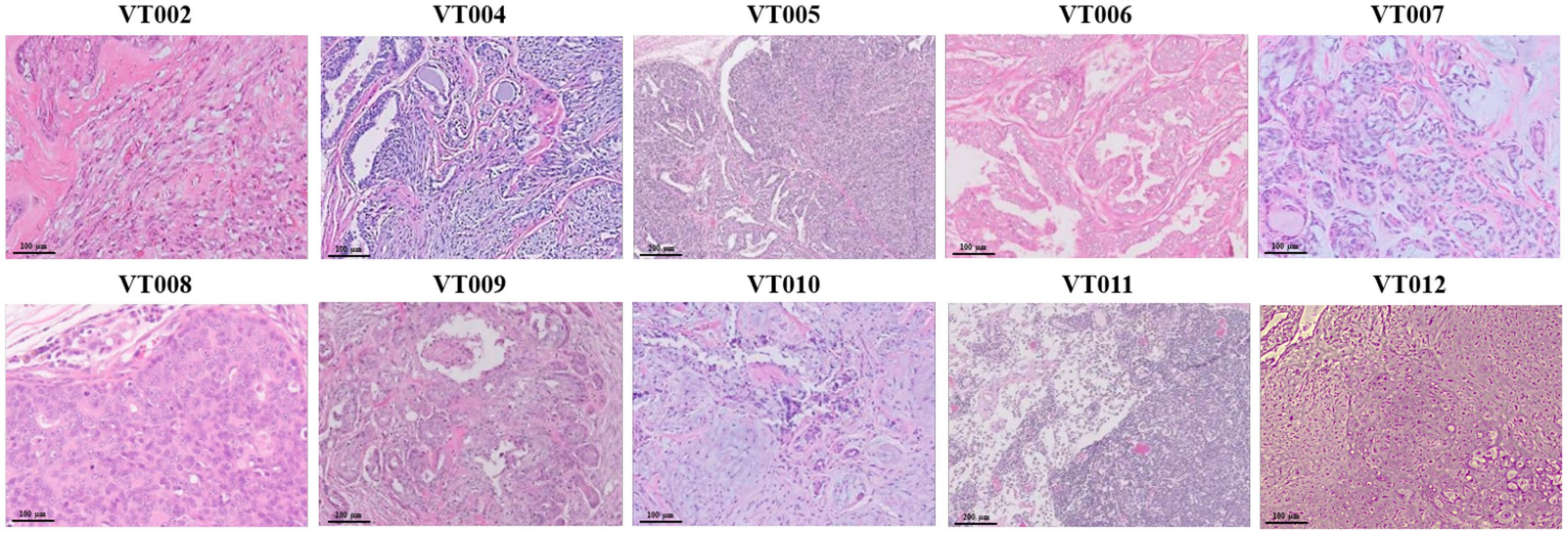
Histopathological examination of VT. Mammary gland mass sectioned and stained with hematoxylin and eosin (10 ×, scale bar = 100 μm).

Canine mammary tumor-derived tissues were obtained from the VIP Animal Medical Center and were kindly gifted by the Haedeun Animal Medical Center. Tumors were classified and diagnosed by veterinary pathologists at the veterinary diagnostic laboratories Neodin Vetlab (Seoul, Korea) and IDEXX Laboratories (Sungnam, Korea). Histopathological examination of VIP tissue (VT) samples was performed using H&E staining. All VTs were graded based on their morphological characteristics, differentiation, proliferative patterns, and invasive growth into the surrounding tissues. The results of these evaluations are presented in Table 2. The tumor cell morphology and phenotype are shown in Figure 1. In VT002, the dermis and subcutis were expanded by a nodular, well-demarcated proliferation of neoplastic mammary epithelial cells and myoepithelial cells surrounded by a dense band of collagenous stroma. The epithelial cells were cuboidal-to-polygonal with indistinct cell borders and moderate amounts of eosinophilic cytoplasm. In VT004, the neoplasm consisted of an intimate admixture of a generally well-organized papillotubular epithelium, myoepithelium, and collagenous-to-myxomatous stroma. In VT005, the epithelial component of the neoplasm was composed of cuboidal to columnar cells arranged in tubules and papillary projections into the tubules, with variable degrees of accumulation on a moderate fibrovascular stroma. In VT006, the specimen was characterized by a well-demarcated and encapsulated proliferation of the mammary gland epithelium. Individual epithelial cells were cuboidal to columnar, with moderate amounts of eosinophilic cytoplasm and mildly pleomorphic euchromatic nuclei. The VT007 cell tissues were composed of two cell populations. The first consists of cuboidal epithelial cells, and the second consists of spindle-to-stellate cells arranged in short interlacing bundles separated by loose collagenous matrix. Multiple foci of cartilaginous and osseous metaplasia were observed throughout the spindle cell population. In VT008, a tumor mass with diffuse, invasive proliferation into the surrounding connective tissue was observed. Differentiation into cartilage and bone tissue was observed within the mass. In VT009, the epithelial neoplasm was interpreted as a carcinoma owing to nuclear pleomorphism, moderate mitotic activity, and invasion into local tissue. At VT010, multiple sections collected from the caudal aspect of the submitted tissue specimen contained moderately hyperplastic lobules of mammary tissue with dilated ducts containing secretions and histiocytes. In VT011, the malignancies included a complex carcinoma, which is a low-grade epithelial malignancy accompanied by benign myoepithelial proliferation and a malignant spindle cell neoplasm. Finally, in VT012 cells, metaplasia and cartilage proliferation were observed in the mammary mass. The non-fixed residual tissues were chopped and cultured for cell isolation. A total of 12 samples were collected for culture; ultimately, 10 samples successfully grew and established cell lines, which we named VIP cell lines (VCLs), corresponding to each VT (Table 2).

**Table 2:** Canine mammary tumor characteristic used to establish each cell line.

Tissue	Cell line	PIK3CA mutation	Breed	Age (years)	Sex	Histologic classification	Doubling time (h)
VT002	VCL002	Mutant	Poodle (Toy)	15	Spayed female	Complex mammary carcinoma, grade I (malignant)	31.1
VT004	VCL004	Mutant	Maltese	10	Female	Benign mixed tumor	22.97
VT005	VCL005	Mutant	Maltese	9	Female	Complex mammary carcinoma, grade I (malignant)	30.44
VT006	VCL006	Wile-type	Welsh Corgi Pembroke	13	Female	Adenoma (benign)	40.03
VT007	VCL007	Mutant	Maltese	14	Female	Benign mixed tumor	22.13
VT008	VCL008	Wild-type	Maltese	15	Female	Malignant mixed mammary tumor	48.57
VT009	VCL009	Mutant	Maltese	14	Spayed female	Complex mammary carcinoma, grade II (malignant)	35.08
VT010	VCL010	Wild-type	Poodle	10	Female	Complex mammary adenoma (benign)	24.42
VT011	VCL011	Wild-type	Maltese	14	Female	Complex mammary carcinoma (malignant)	25.51
VT012	VCL012	Wild-type	Mixed breed	13	Spayed female	Benign mixed tumor	40.73

### Sequence analysis of PIK3CA mutation

**Figure 2:**
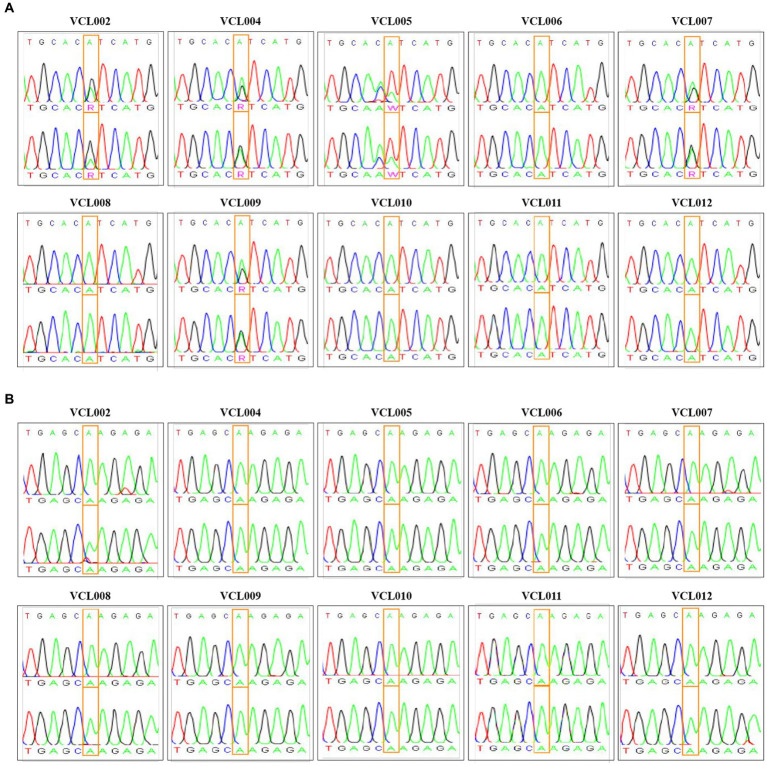
PIK3CA mutation profile in VCL. **(A)** Normal samples exhibited homozygous 3140A (1047His) peak (indicated as green ′A′ in the box); VCL006, VCL008, VCL010, VCL011, and VCL012. Mutant samples exhibited heterozygous 3140A/G (H1047R) peak (indicated as pink ′R′ in the box); VCL002, VCL004, VCL007, and VCL009. Another mutant sample exhibited heterozygous 3140A/T (H1047L) peak (indicated as pink ′W′ in the box); VCL005. **(B)** PIK3CA Q546P (c.1637 A > C) mutation was detected by using Sanger sequencing.

To investigate the frequency of PIK3CA mutation in CMT, gDNA extracted from each VCL and the amplification products were analyzed by Macrogen (Seoul, Korea). Based on previous studies (23, 24), we focused on two variants known to exactly match the mutation spots related to human breast cancer. The PIK3CA mutation analysis was performed using Sanger sequencing to detect the c.3140A > G (H1047R) and c.1637A > C (Q546P) mutations. The sequence data from this study has been submitted to the NCBI Sequence Read Archive (SRA accession number: SRR26661417-SRR26661456). Using the PIK3CA 3,140 primer set, amplification of the flanking regions of nucleotide positions 1,540–1,659 detected that exon 20 of PIK3CA (A3140G) mutation was found in 50% (5/10) of the VCL cases (Figure 2A). Specifically, VCL006, VCL008, VCL010, VCL011, and VCL012 exhibited a homozygous 3140A (1047H) peak. However, VCL002, VCL004, VCL007, and VCL009 exhibited a heterozygous 3140A/G (H1047R) peak, resulting in a change of the 1047th amino acid from Histidine (His) to arginine (Arg). Only VCL005 sample showed a heterozygous 3140A/T (H1047L) peak, changing the 1047th amino acid from His to Leucine (Leu). However, using the PIK3CA 1,637 primer set, amplifying the flanking regions of nucleotide positions 3,023–3,207, another PIK3CA (A1637C) mutation was not found in this study (Figure 2B).

### Characteristic analysis of established VCLs

**Figure 3:**
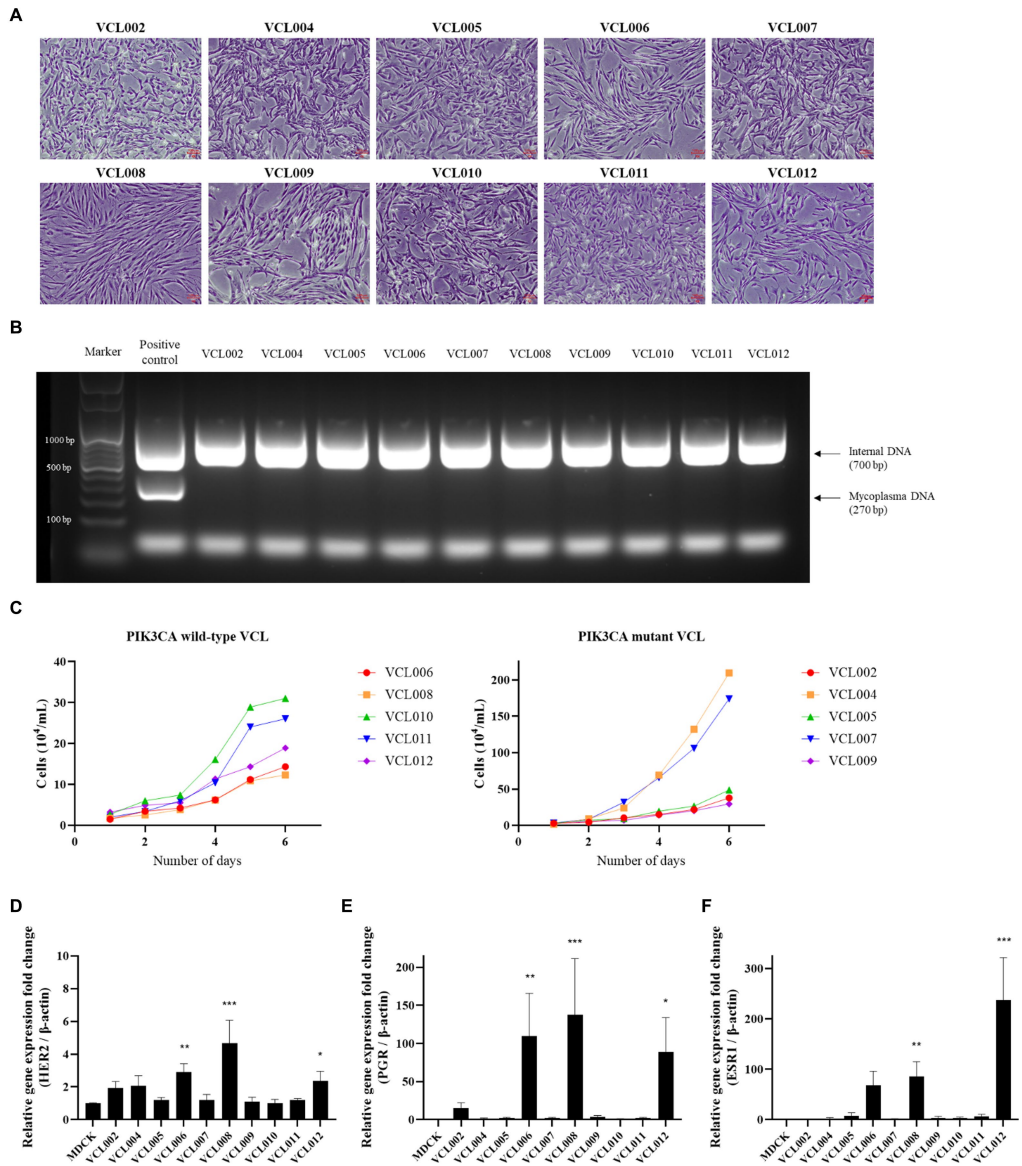
Characteristic analysis of VCLs. **(A)** Morphology of canine mammary cancer cell line established from mammary tumor tissue (4 ×, scale bar = 100 μm). **(B)** DNA extracted from cell cultured media and mycoplasma contamination test was conducted by PCR. No mycoplasma was detected in all VCLs. **(C)** Growth curves of PIK3CA wild-type cell lines and PIK3CA mutant cell lines. **(D–F)** mRNA expressions of canine HER2, PGR, and ESR1 in VCLs. β-actin was used as a reference gene and normal canine MDCK cell line was used as control. The values indicate the mean ± standard deviation (SD) of three independent experiments performed in triplicates, and statistical significances are indicated as *p < 0.05, **p < 0.01, and ***p < 0.001.

We further analyzed the characteristics of VCLs, such as their cell morphology, growth rate, and gene expression, to investigate whether the PIK3CA mutation status is related to the features of canine mammary tumors. Information for each canine patients and the features of the VCL are shown in Table 2. At the beginning of culture, pleomorphism of cells was observed; however, as the number of passages increased through subculture, the shape of the cells tended to remain constant, and the presence of fibroblasts decreased because of the trypsin method. Most cells exhibited epithelioid and spindle shape (Figure 3A). Mycoplasma contamination was not detected in any VCL samples (Figure 3B). Cell growth was generally faster in the PIK3CA mutant group than that in the wild-type (Figure 3C). The doubling time of wild-type VCL was usually over 40 h, except for VCL010 and VCL011, which had doubling times of 24.42 h and 25.51 h, respectively. In the PIK3CA mutant group, VCL004 and VCL007 had doubling times calculated as 22.97 h and 22.13 h, respectively, whereas those of most of the others were approximately 30 h. The mRNA expression levels of genes related to breast cancer are shown in Figures 3D–F. Compared with the normal canine MDCK cell line, HER2, also known as Erb-b2 receptor tyrosine kinase 2 (ERBB2), expression was significantly increased in the three wild-type VCL (VCL006, VCL008, and VCL012). Similarly, the expression of progesterone receptor (PGR) and estrogen receptor 1 (ESR1) dramatically increased in VCL006, VCL008, and VCL012. However, in other wild-type or mutant cell lines, the relative gene expression levels of the three genes remained similar to those in the normal canine cell line (Figures 3D–F).

### Cytotoxic effect of alpelisib on the proliferation of VCLs and relevance to PIK3CA mutation

**Figure 4:**
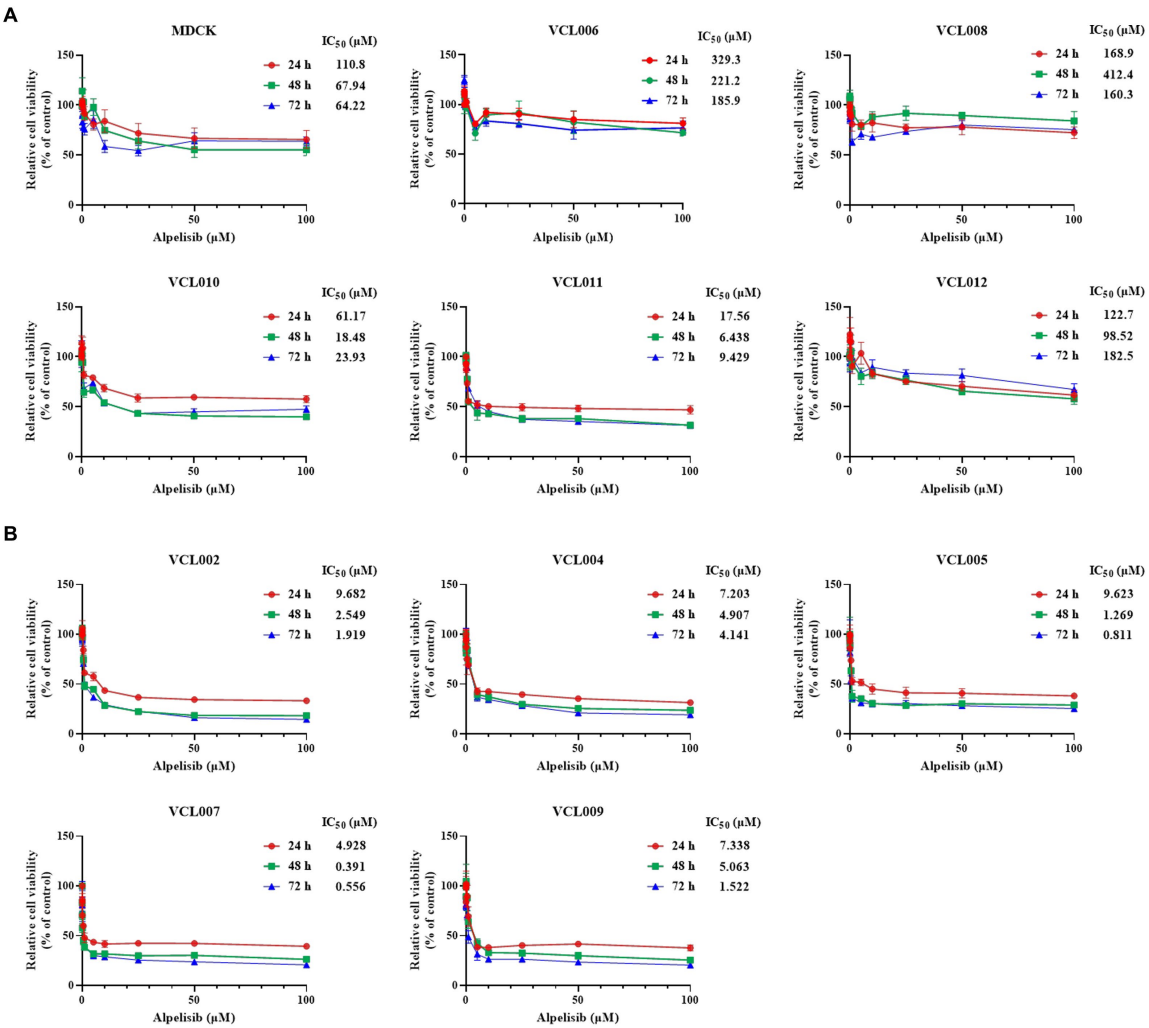
Cytotoxicity effect of alpelisib on VCLs. Each cell lines were treated with various concentrations of alpelisib (0, 0.01, 0.05, 0.1, 0.5, 1, 5, 10, 25, 50, and 100 μM) for 24, 48, and 72 h. The cell viability was measured by using WST assay kit and the IC_50_ values (μM) were calculated. **(A)** The IC_50_ values and cell viabilities of normal canine MDCK cell line and PIK3CA wild-type VCLs. **(B)** The IC_50_ values and cell viabilities of PIK3CA mutant VCLs. The values indicate the mean ± standard deviation (SD) of three independent experiments performed in triplicates.

To investigate the effect of alpelisib, a PI3K alpha-specific inhibitor, on the viability of CMT cells, VCLs were treated with various concentrations of alpelisib for 24, 48, and 72 h. In all VCL, the viabilities of cells treated with alpelisib for 24, 48, and 72 h. In all VCLs, the viability of cells treated with alpelisib for 24, 48, and 72 h significantly decreased in a time- and dose-dependent manner (Supplementary Tables S1–S3). However, the IC_50_ values in the PIK3CA-mutant cell lines were markedly lower than those in the PIK3CA-wild-type cell lines (Figure 4). PIK3CA-mutant VCLs were more sensitive to alpelisib than wild-type ones. For 24 h, the IC_50_ values of PIK3CA-mutant VCLs were calculated to be 9.682 μM (VCL002), 7.203 μM (VCL004), 9.623 μM (VCL005), 4.928 μM (VCL007), and 7.338 μM (VCL009). Meanwhile, the IC_50_ values of the wild-type cell lines were above 100 μM, except for VCL010 and VCL011, which showed relatively low values. The IC_50_ value of the normal canine cell line, without to the PIK3CA mutation, was also higher than that of the mutant cell lines.

### Inhibitory effect of alpelisib on the migration of VCL and relevance to the PIK3CA mutation

**Figure 5:**
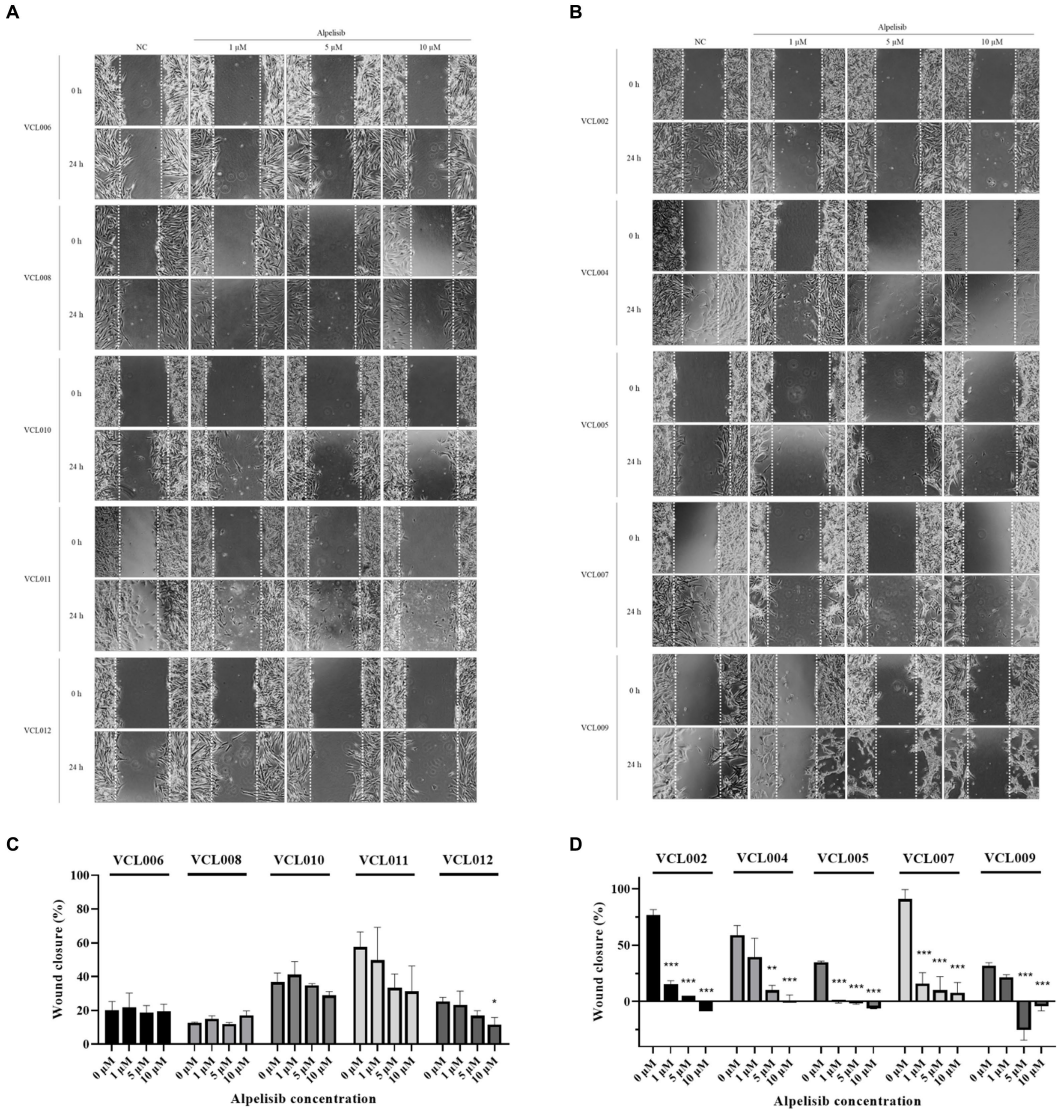
The migration of VCLs was measured by the wound healing assay after formation of scratch and 24 h treatment of various concentrations of alpelisib (0, 1, 5, and 10 μM). **(A)** Representative images of the scratched areas of PIK3CA-wild-type VCLs were taken at 0 and 24 h. **(B)** Representative images of the scratched areas of PIK3CA-mutant VCLs were taken at 0 and 24 h. **(C)** Wound (scratch) closure of PIK3CA-wild-type VCLs and **(D)** wound closure of PIK3CA-mutant VCLs were quantified using Image J software. The values indicate the mean ± standard deviation (SD) of three independent experiments performed in triplicates, and statistical significances are indicated as *p < 0.05, **p < 0.01, and ***p < 0.001.

In the normal canine cell line, alpelisib treatment did not affect cell migration, except at the highest concentration for 24 h (Supplementary Figure S1), and similar aspects of PIK3CA-wild-type VCLs were observed (Figure 5A). The scratched areas of the cells treated with a low dose of alpelisib did not show significant differences. Meanwhile, in PIK3CA-mutant VCLs, the wound closure of cells treated with alpelisib for 24 h showed significant inhibition of cell migration compared to the non-treated cell (Figure 5B). After 24 h, a few scratched areas were wider than those before treatment in cells treated with a high concentration of alpelisib (Figures 5C,D). Because of the long doubling time of certain cell lines, 48 h treatment of alpelisib was also conducted. The wound closure of PIK3CA-mutant VCLs was found to be inhibited by alpelisib treatment, whereas those of PIK3CA-wild-type VCLs was not inhibited even when treated with alpelisib (Supplementary Figure S2). These results also showed that PIK3CA-mutant VCLs were more responsive to alpelisib than wild-type VCLs.

### Inhibitory effect of alpelisib on GLUT mRNA expression and relevance to the PIK3CA mutation

**Figure 6:**
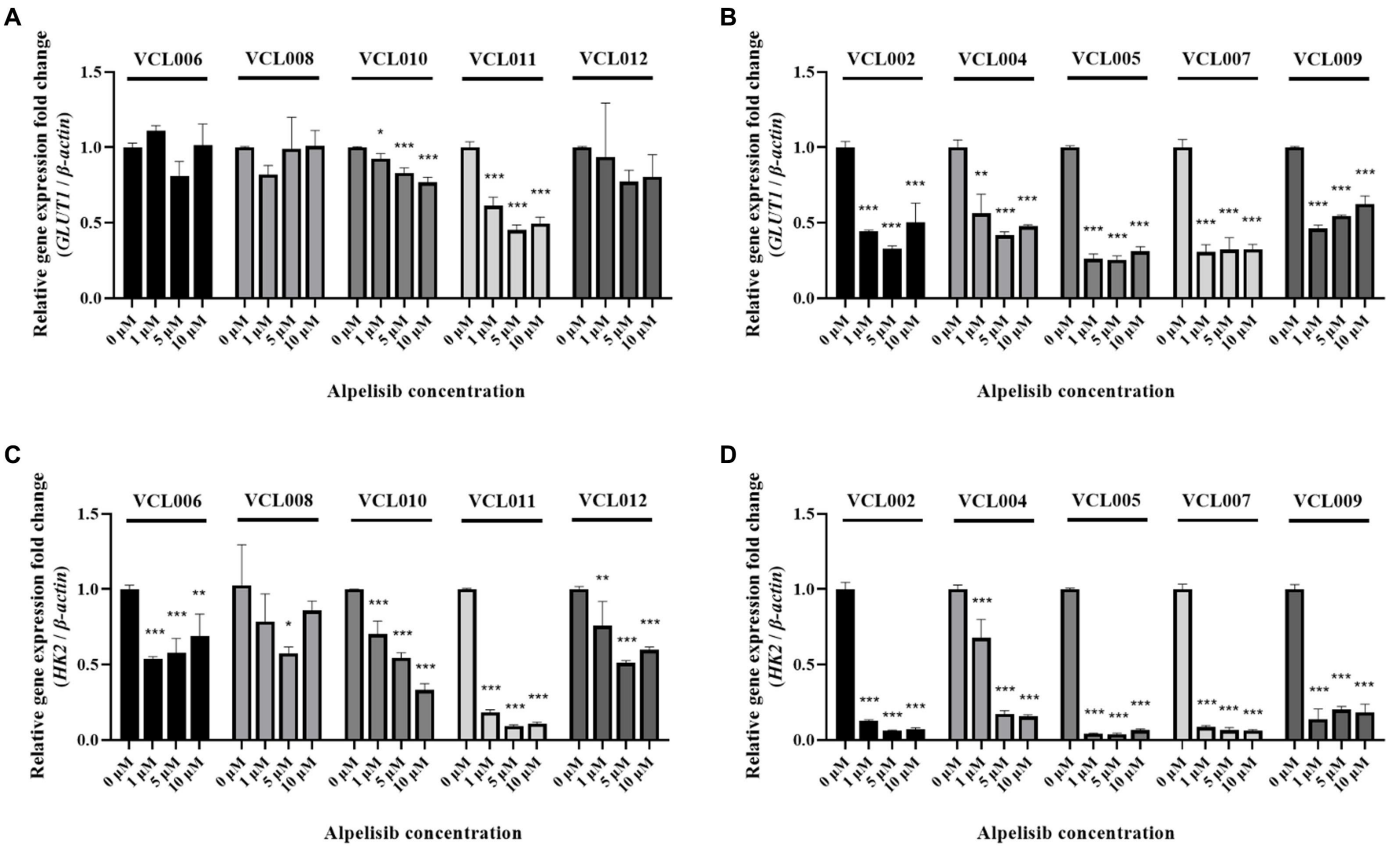
The mRNA levels of glucose metabolism-related genes (GLUT1, HK2) were measured by qRT-PCR after 24 h treatment of various concentrations of alpelisib (0, 1, 5, and 10 μM). **(A)** The expression level of canine GLUT1 in PIK3CA-wild-type VCLs. **(B)** The expression level of canine GLUT1 in PIK3CA mutant VCLs. **(C)** The expression level of HK2 in PIK3CA-wild-type VCLs. **(D)** The expression level of canine HK2 in PIK3CA-mutant VCLs. β-actin was used as a reference gene. The values indicate the mean ± standard deviation (SD) of three independent experiments performed in triplicates, and statistical significances are indicated as *p < 0.05, **p < 0.01, and ***p < 0.001.

To examine whether alpelisib-induced tumor cell death and migration inhibition also affected gene expression, we performed qRT-PCR. The mRNA expression of glucose transporter 1 (GLUT1), also known as solute carrier family 2, facilitated glucose transporter member 1 (SLC2A1), which regulates the uptake of glucose in most cell types, was significantly decreased by alpelisib treatment in PIK3CA-mutant cell lines (Figure 6B). Even a 1 μM alpelisib treatment effectively inhibited GLUT1 mRNA level to 0.442- (VCL002), 0.564- (VCL004), 0.262- (VCL005), 0.307- (VCL007) and 0.462-fold (VCL009). The mRNA level of hexokinase 2 (HK2), which catalyzes glucose metabolism, was also significantly downregulated by alpelisib in the same group (Figure 6D). However, in PIK3CA-wild-type cell lines, fold-changes in GLUT1 and HK2 gene expression were slightly decreased with alpelisib treatment compared to the mutant groups (Figures 6A,C). Only VCL010 and VCL011, which exhibited particularly low IC_50_ value in the wild-type VCLs, showed similar mRNA expression levels as the mutant group.

## Discussion

Mammary cancer is common in both dogs and humans. Canine mammary tumors occur at a high rate in intact female dogs, which is one reason why the majority of female dogs undergoing spaying surgeries. However, according to several studies, the preventive effect on mammary tumors is only observed when spaying is performed at a young age, and spaying female dogs over the age of 2 years does not provide any benefit in preventing mammary cancer (29, 30). Mammary tumors are a significant disease in female dogs, and most cases are treated with mammary gland excision. However, recurrence and metastasis remain significant concerns in these animals (31). Therefore, there is a need for new drugs that can inhibit the growth and activity of canine mammary tumors. Here, we investigated a new treatment approach for CMT thusly: First, we confirmed the incidence of PIK3CA mutations in CMT cases, and the anticancer effect of a PIK3CA inhibitor was investigated by establishing novel canine cell lines from tumor tissues based on the presence of the mutation.

Many studies have suggested that PIK3CA is one of the most frequently mutated genes in breast cancer, especially in the hormone receptor-positive (HR+)/HER2- subgroup (15, 18–20). Although few CMT samples were investigated in the present study, we found that some PIK3CA mutations occurred that closely matched those observed in human breast cancers. In the mutation analysis of 10 cases of canine mammary tumors, five cases showed a missense mutation at the 1047th amino acid in exon 20. Among them, four cases exhibited the H1047R heterozygous mutation, in which His had been changed to Arg, and one case exhibited a H1047L heterozygous mutation, in which His had been changed to Leu. However, in this study, no PIK3CA mutations were found in another hotspot in exon 9. These results suggested that PIK3CA mutations frequently occur in CMT, and as mentioned in other studies (23–27), PIK3CA mutations may be highly relative to CMT. Canine mammary tumors can appear as both benign and malignant tumors. In our study, half of the canine mammary tumors were benign, whereas the remainder were malignant. We expected PIK3CA mutations to occur at a higher frequency in malignant tumors. However, there was no apparent relationship between the occurrence of PIK3CA mutations and malignant or benign tumors. Among the five mutant cases, three tumors were classified as malignant; however, even the wild-type tumors showed a similar ratio of malignant tumors (two malignant and three benign). This suggests that PIK3CA mutations may play a major role in regulating cell proliferation at an early stage, and that benign tumors may progress to malignancy through additional factors or mutations (32). It appears that there is no direct relationship between PIK3CA mutations and tumor malignancy; however, we believe that further investigation is necessary through follow-up studies.

There are several established canine cell lines derived from mammary cancer, such as CMT-U27, CMT-12, and CMT-7364 (33–35). However, there is currently no canine cell line that specifically focuses on the novel breast cancer-associated mutational gene, PIK3CA. While other studies have developed various canine cell lines originating from mammary tumors (3–5), PIK3CA mutation in these established cell lines are yet to be characterized. Therefore, we established novel CMT cell lines that we named VCL. These cell lines were characterized by the presence of PIK3CA c.3140 A > G (H1047R) and c.3140 A > T (H1047L) mutations, similar to other PIK3CA-mutant CMT cell lines. All the VCLs exhibited similar sizes and morphologies, but their doubling times varied. VCL007 had the shortest doubling time (22.13 h), whereas VCL008 had the longest (48.57 h). Generally, PIK3CA mutant VCL cells grew rapidly and had shorter doubling times than wild-type cells. PIK3CA wild-type VCL cells generally had a doubling time of 40 h or more, except for VCL010 and VCL011, which showed values similar to those of the mutant cells. Furthermore, mRNA expression analysis of three breast cancer markers (HER2, PGR, and ESR1) revealed relatively high expression in VCL006, VCL008, and VCL012 compared to other cell lines. Besides these lines, most VCLs exhibited similar levels of gene expression to those of the normal canine cell line. In contrast to previous studies on human breast cancer (36, 37), we observed a higher frequency of PIK3CA mutations in patients with low ESR1 and PGR expression. However, there is a limitation in considering the HER2, PGR, and ESR1 expression of each cell line as accurate, as they were compared with cell lines derived from different tissues of healthy dogs. Therefore, additional experiments are required to compare gene expression by establishing a cell line from the mammary tissues of healthy female dogs. Here, the characteristics of the newly established VCLs were observed only at the mRNA level; protein levels were not measured. Therefore, to use established cell lines, further experiments, such as immunocytochemistry or western blotting, should be conducted to classify the definite characteristics of each cell.

Alpelisib is a specific inhibitor designed to target and block the PI3Kα isoform. Alpelisib has been developed and used due to the association between human breast cancer and PIK3CA mutations (38, 39). However, the use of alpelisib in dogs has not yet been generalized. In another study, alpelisib treatment was used to confirm whether CMT-derived organoids were properly generated, and the results varied depending on the presence of PIK3CA mutations (40). In the present study, the analysis of anticancer effect of alpelisib on VCLs demonstrated that PIK3CA mutant cells were more sensitive and responsive to alpelisib treatment than PIK3CA wild-type cells. These results are consistent with those of a previous study demonstrating the anticancer effect of alpelisib against PIK3CA-mutated canine hemangiosarcoma cells (23). Even at low concentration (< 10 μM), alpelisib significantly inhibited cell proliferation, particularly in the PIK3CA mutant cell lines. Conversely, the viabilities of normal canine cells and PIK3CA wild-type cells were reduced with high-concentrations of alpelisib (> 100 μM) treatment for 24 h, except for VCL010 and VCL011, which exhibited rapid growth rates. These results suggest that alpelisib, a novel therapy for human breast cancer, may also hold potential as a drug for CMT treatment, and could be particularly effective in tumors with PIK3CA mutation.

In addition to the migration analysis, low-dose alpelisib treatment significantly suppressed cell migration in PIK3CA-mutated cell lines. At the highest concentration of alpelisib, the scratch closure was wider than the initial area owing to toxicity. In contrast, alpelisib did not inhibit the migration of PIK3CA wild-type VCLs. Except for the rapidly growing cells, it can be observed that in the control group, there is no notable difference in the scratch area after 24 h of treatment owing to the low migration rate of these cells. Therefore, it may be challenging to precisely analyze the effects of alpelisib administration. However, when comparing the cell lines that exhibited swift migration ability in the non-treated state, such as VCL002, VCL007, VCL011, and MDCK, the percentage of wound closure was significantly reduced by low-concentration alpelisib treatment in PIK3CA mutants but not in wild-type VCLs. For slow-growing cell lines, the addition of 48 h experiment showed a remarkable difference between PIK3CA mutant VCLs and PIK3CA wild-type VCLs. In the PIK3CA mutant cell line, a high migration rate was observed in the non-treated cells, whereas in the alpelisib-treated cells, migration was significantly suppressed. Meanwhile, the wound closure in wild-type cells and normal canine cells decreased only when treated with a high concentration of alpelisib. These findings suggest that alpelisib effectively suppresses the migration of PIK3CA-mutated tumor cells. However, because the concentration of alpelisib used in the wound healing assay of this study is relatively high, it could potentially affect not only cell migration but also cell death. Therefore, it is necessary to conduct additional experiments using a lower concentration of the drug and transwell system.

Since the PI3K signaling pathway plays a crucial role in energy generation for the survival of cancer cells (41–43), the effect of alpelisib on the gene expression to glucose metabolism-related genes was analyzed using qRT-PCR. Following alpelisib treatment, the mRNA level of GLUT1, a known glucose transporter, was significantly reduced in PIK3CA mutant VCLs. Furthermore, the mRNA level of HK2, a glycolytic enzyme that regulates the first step of glycolysis, was significantly reduced in mutant VCLs. However, the mRNA level in the wild-type groups did not decrease, except for VCL010 and VCL011, as evidenced by their IC_50_ values, which were the lowest observed among the wild-type cells. These results suggested that alpelisib inhibited the glucose metabolism of PIK3CA-mutated cells.

Overall, our study revealed that alpelisib is effective against PIK3CA-mutant CMT, similar to its effectiveness against PIK3CA-mutated human breast cancer. Alpelisib may be used more effectively than other drugs in CMT patients when there is a concurrent occurrence of a PIK3CA mutation and a malignant diagnosis, similar to its use to prevent recurrence and metastasis in metastatic human breast cancers with PIK3CA-mutations. Alpelisib could be utilized in the treatment of PIK3CA-mutated benign tumors when surgical excision is not feasible owing to factors such as old age or dog owner’s reluctance. Additionally, we successfully established CMT cell lines characterized by the presence or absence of PIK3CA mutation. Given the similarity between canine and human breast cancers, the cell lines established in this study could be useful for the development of new drugs targeting PIK3CA mutation-derived human breast cancer. However, it is important to acknowledge the limitations of our study, which include a low number of CMT samples analyzed and the lack of investigation into PIK3CA mutation in the normal canines. Further investigations are needed to gain a more comprehensive understanding of CMT. Another limitation is that the growth rate varies among individual cell lines. Because this factor could potentially impact the interpretation of the results, we believe it is necessary to conduct experiments to determine if this changes the outcomes under controlled conditions. Additionally, for cell lines such as VCL008 and VCL012, where chondrocytes might have been mixed with mammary epithelial cells, it would be necessary to further purify the cells through processes such as flow cytometry before they can be used in other studies.

## Conclusion

In conclusion, this study investigated PIK3CA H1047 and Q546 mutations in CMT and established novel canine cell lines with characteristics PIK3CA mutations. We also demonstrated that alpelisib successfully inhibits tumor cell viability, cell migration, and gene expression related to glucose uptake in canine mammary cells. These findings suggest that the PIK3CA H1047 mutation could serve as a useful prognostic biomarker for CMT, and alpelisib may be a potential drug for targeting PIK3CA-mutant CMT. Further studies are required to investigate the mechanism of action of alpelisib in CMT.

## Data availability statement

The original contributions presented in the study are publicly available. This data can be found here: https://www.ncbi.nlm.nih.gov/sra/, acession numbers SRR26661417-SRR26661456.

## Ethics statement

The animal studies were approved by VIP Research Institute, VIP Advanced R&D: IACUC NON 2022-0002. The studies were conducted in accordance with the local legislation and institutional requirements. Written informed consent was obtained from the owners for the participation of their animals in this study.

## Author contributions

JY: Conceptualization, Data curation, Formal analysis, Funding acquisition, Investigation, Methodology, Project administration, Resources, Software, Supervision, Validation, Visualization, Writing – original draft, Writing – review & editing. YC: Funding acquisition, Project administration, Supervision, Writing – original draft. SA: Funding acquisition, Project administration, Resources, Supervision, Writing – original draft. SJ: Conceptualization, Investigation, Resources, Writing – original draft.

## Footnotes

^1^ http://www.doubling-time.com/compute.php
