# Supplementary material for: Anticancer effects of alpelisib on PIK3CA-mutated canine mammary tumor cell lines
Source: Front Vet Sci. 2023 Nov 15;10:1279535. doi: 10.3389/fvets.2023.1279535 (PMC10684731; doi:10.3389/fvets.2023.1279535)
Supplement: Supplementary file 1 [file Data_Sheet_1.pdf]

Supplementary Material

1 Supplementary Tables

Supplementary Table 1. Cytotoxicity of alpelisib against VCLs for 24 h.

Alpelisib (μM)	VCL002	VCL004	VCL005	VCL006	VCL007	VCL008	VCL009	VCL010	VCL011	VCL012
**0**	99.974 ± 5.659	100.024 ± 4.334	99.971 ± 9.420	100.000 ± 2.859	100.023 ± 1.641	99.924 ± 2.971	100.000 ± 3.097	100.018 ± 3.949	100.015 ± 1.908	100.055 ± 4.212
**0.01**	103.279 ± 2.211	95.528 ± 8.109	86.018 ± 7.095	113.103 ± 5.413	86.059 ± 6.255	97.945 ± 5.398	83.792 ± 4.562	101.524 ± 3.532	92.834 ± 7.140	115.525 ± 7.338
**0.05**	99.010 ± 3.550	93.192 ± 11.953	94.057 ± 3.448	113.333 ± 2.700	83.262 ± 6.166	93.455 ± 0.923	98.197 ± 11.355	107.787 ± 4.236	92.714 ± 5.084	119.144 ± 9.527
**0.1**	106.204 ± 7.651	87.100 ± 18.090	98.668 ± 6.795	110.345 ± 3.128	70.399 ± 9.513	90.868 ± 4.718	101.989 ± 13.086	113.664 ± 7.455	87.377 ± 3.345	122.451 ± 16.909
**0.5**	84.225 ± 3.933	75.236 ± 15.640	73.832 ± 2.394	100.805 ± 3.814	59.933 ± 2.751	89.193 ± 3.719	89.619 ± 11.043	108.650 ± 10.888	73.288 ± 2.190	114.903 ± 13.825
**1**	61.252 ± 2.809	69.381 ± 0.643	52.954 ± 3.770	102.492 ± 3.720	47.912 ± 4.876	80.897 ± 7.077	69.402 ± 9.693	81.642 ± 3.602	55.480 ± 1.345	90.328 ± 7.087
**5**	57.518 ± 4.172	42.814 ± 3.427	51.707 ± 3.278	80.575 ± 2.322	43.569 ± 1.545	80.213 ± 4.845	38.382 ± 0.992	79.008 ± 1.889	52.048 ± 3.987	103.463 ± 10.996
**10**	43.612 ± 2.020	42.524 ± 2.830	45.086 ± 5.114	92.069 ± 4.353	41.677 ± 3.413	82.135 ± 9.185	38.167 ± 2.190	68.612 ± 3.749	50.286 ± 2.145	83.224 ± 3.990
**25**	36.678 ± 1.693	39.512 ± 1.522	41.340 ± 5.345	90.257 ± 6.089	42.394 ± 2.277	77.262 ± 3.798	40.315 ± 1.856	58.741 ± 4.079	49.307 ± 3.794	75.191 ± 2.849
**50**	34.436 ± 1.693	35.476 ± 0.835	40.778 ± 4.604	84.894 ± 8.281	42.313 ± 1.517	78.036 ± 7.773	41.676 ± 1.898	59.456 ± 2.288	48.238 ± 3.322	70.383 ± 4.671
**100**	33.368 ± 1.987	31.488 ± 1.287	38.228 ± 2.008	81.118 ± 5.430	39.398 ± 1.931	73.312 ± 5.694	37.809 ± 2.977	57.565 ± 3.526	46.868 ± 4.256	61.530 ± 5.095

Supplementary Table 2. Cytotoxicity of alpelisib against VCLs for 48 h.

Alpelisib (μM)	VCL002	VCL004	VCL005	VCL006	VCL007	VCL008	VCL009	VCL010	VCL011	VCL012
**0**	99.989 ± 2.771	100.000 ± 3.964	99.987 ± 17.155	100.080 ± 9.313	100.009 ± 0.653	99.909 ± 0.316	100.015 ± 1.791	100.000 ± 3.020	99.987 ± 1.381	99.950 ± 7.343
**0.01**	105.309 ± 3.942	90.209 ± 3.462	98.156 ± 4.287	109.829 ± 1.880	83.265 ± 3.433	106.393 ± 3.954	89.443 ± 6.402	94.428 ± 9.640	101.497 ± 2.556	101.702 ± 13.024
**0.05**	101.519 ± 3.767	86.092 ± 7.942	95.247 ± 8.058	111.257 ± 4.168	70.429 ± 4.237	109.224 ± 2.604	100.442 ± 12.270	102.425 ± 4.209	96.966 ± 6.718	99.652 ± 12.201
**0.1**	97.025 ± 2.092	81.366 ± 6.001	89.210 ± 4.771	106.457 ± 5.057	57.837 ± 4.557	106.210 ± 8.648	104.821 ± 16.921	102.440 ± 10.208	95.376 ± 3.031	101.315 ± 15.189
**0.5**	74.543 ± 4.185	84.058 ± 6.194	63.447 ± 2.996	98.000 ± 3.084	44.776 ± 3.823	95.799 ± 3.175	87.933 ± 12.986	94.111 ± 8.956	77.530 ± 1.115	105.493 ± 16.466
**1**	48.183 ± 3.795	72.952 ± 2.218	37.640 ± 6.015	97.356 ± 3.937	38.324 ± 1.114	92.031 ± 5.185	64.027 ± 6.827	67.421 ± 5.134	54.756 ± 1.968	92.903 ± 5.567
**5**	44.716 ± 1.134	39.462 ± 4.796	35.269 ± 2.647	71.086 ± 6.970	31.847 ± 3.284	79.087 ± 2.214	42.533 ± 4.131	66.521 ± 2.399	43.680 ± 7.179	80.271 ± 8.174
**10**	28.612 ± 2.033	37.222 ± 4.195	30.621 ± 3.009	89.503 ± 5.981	31.819 ± 1.446	88.047 ± 5.272	33.065 ± 2.556	53.875 ± 2.038	42.991 ± 1.352	83.077 ± 5.065
**25**	22.379 ± 1.131	29.716 ± 2.842	28.588 ± 2.059	91.667 ± 11.759	29.964 ± 0.679	91.837 ± 7.159	32.503 ± 3.031	43.296 ± 1.990	37.918 ± 3.222	76.576 ± 4.360
**50**	18.557 ± 1.374	25.575 ± 1.818	30.358 ± 2.521	82.212 ± 11.421	30.358 ± 1.158	89.504 ± 3.362	29.970 ± 3.447	40.746 ± 1.263	38.011 ± 1.851	65.608 ± 3.770
**100**	18.293 ± 1.339	23.664 ± 2.772	29.090 ± 2.362	71.554 ± 3.406	26.281 ± 1.269	84.159 ± 9.385	25.588 ± 2.086	39.863 ± 2.985	31.462 ± 1.098	57.767 ± 5.423

Supplementary Table 3. Cytotoxicity of alpelisib against VCLs for 72 h.

Alpelisib (μM)	VCL002	VCL004	VCL005	VCL006	VCL007	VCL008	VCL009	VCL010	VCL011	VCL012
**0**	100.024 ± 2.384	99.991 ± 1.782	99.976 ± 14.666	99.856 ± 3.151	99.979 ± 4.710	99.891 ± 6.082	100.000 ± 2.072	99.986 ± 5.262	100.012 ± 0.458	99.875 ± 7.613
**0.01**	96.077 ± 5.926	102.570 ± 3.452	95.068 ± 4.004	124.854 ± 4.549	81.013 ± 7.276	88.358 ± 2.550	80.537 ± 3.894	97.589 ± 8.280	97.072 ± 5.287	93.642 ± 9.005
**0.05**	94.751 ± 6.627	92.309 ± 6.413	86.721 ± 2.992	123.333 ± 4.480	74.487 ± 6.530	85.871 ± 4.491	81.729 ± 4.304	109.196 ± 6.728	101.635 ± 2.946	99.457 ± 6.641
**0.1**	95.329 ± 3.947	91.084 ± 5.014	81.687 ± 4.055	111.404 ± 5.756	71.222 ± 6.786	91.741 ± 1.799	80.698 ± 5.008	103.883 ± 12.165	99.353 ± 2.037	105.304 ± 9.748
**0.5**	70.700 ± 2.652	83.777 ± 10.347	54.080 ± 4.381	98.830 ± 3.863	47.901 ± 2.664	88.557 ± 4.370	70.741 ± 4.855	97.289 ± 3.443	89.239 ± 2.474	104.026 ± 8.626
**1**	48.859 ± 3.147	70.941 ± 5.325	36.364 ± 3.661	98.851 ± 2.516	38.843 ± 2.055	62.963 ± 3.140	48.808 ± 6.255	68.378 ± 5.338	68.311 ± 0.946	99.687 ± 6.549
**5**	36.644 ± 1.361	36.927 ± 3.914	31.150 ± 1.199	77.135 ± 6.242	30.149 ± 3.415	70.945 ± 5.086	31.408 ± 6.168	73.842 ± 1.672	51.025 ± 4.692	83.930 ± 2.655
**10**	29.358 ± 2.261	34.402 ± 2.589	30.145 ± 3.167	83.405 ± 5.280	28.822 ± 0.842	67.865 ± 2.929	26.439 ± 0.751	53.990 ± 3.236	45.189 ± 2.769	89.404 ± 7.426
**25**	22.747 ± 1.962	28.438 ± 2.089	30.618 ± 3.023	80.819 ± 3.964	25.615 ± 1.419	73.638 ± 3.224	26.380 ± 1.422	43.492 ± 2.973	37.347 ± 1.402	83.574 ± 3.381
**50**	16.266 ± 1.374	21.114 ± 0.603	28.279 ± 2.682	74.210 ± 9.046	23.764 ± 0.688	80.065 ± 4.323	23.591 ± 2.714	44.784 ± 3.149	35.187 ± 2.270	81.254 ± 6.460
**100**	14.507 ± 1.395	19.169 ± 0.625	25.394 ± 2.672	76.437 ± 6.988	20.789 ± 0.990	75.490 ± 2.143	20.507 ± 0.156	47.417 ± 3.426	31.149 ± 1.640	67.210 ± 5.723

2 Supplementary Figures

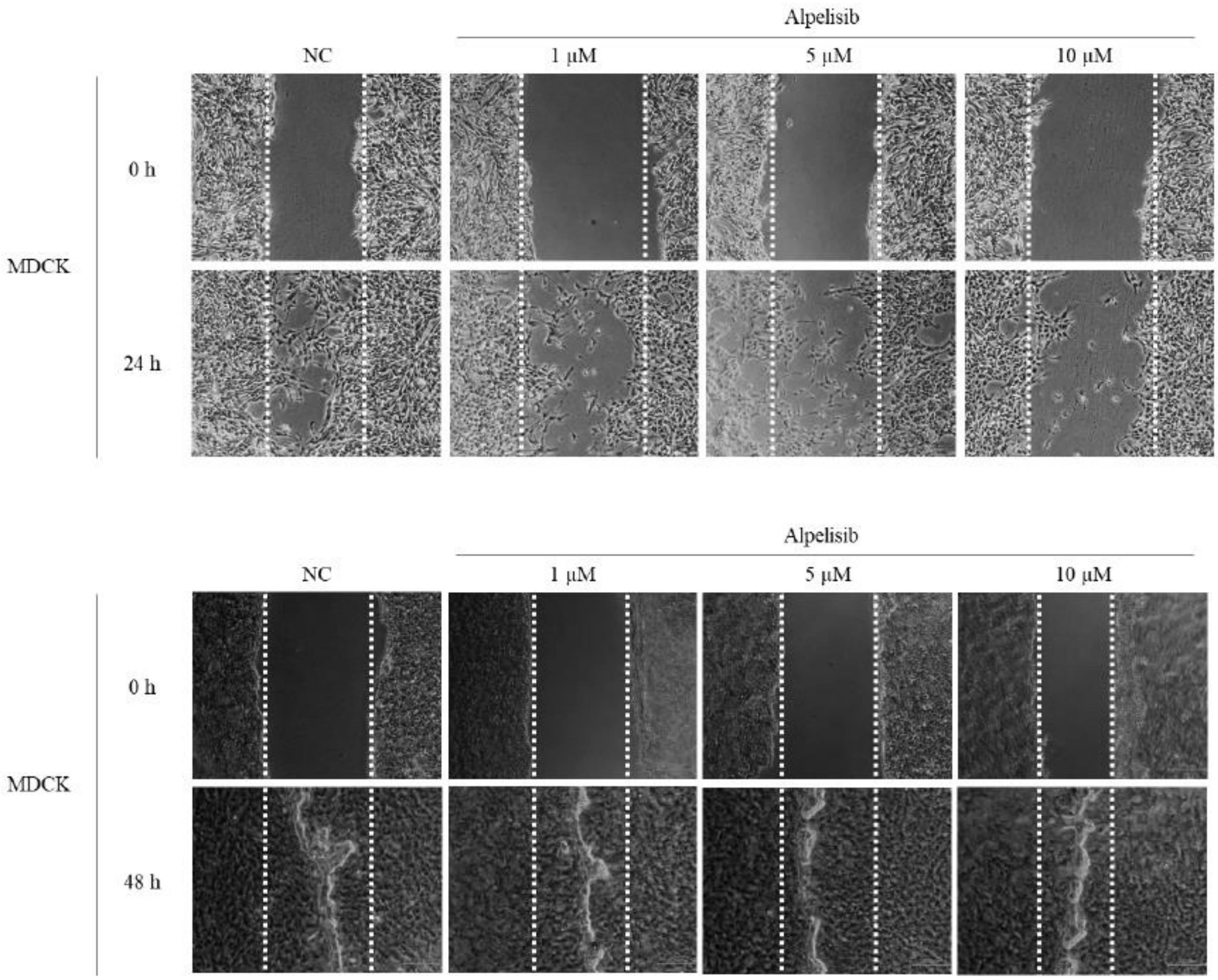

Supplementary Figure 1. The migration of MDCK were measured by the wound healing assay. After formation of scratch, the cells were treated with various concentrations of alpelisib (0, 1, 5, and 10 μM) for 24 h and 48 h.

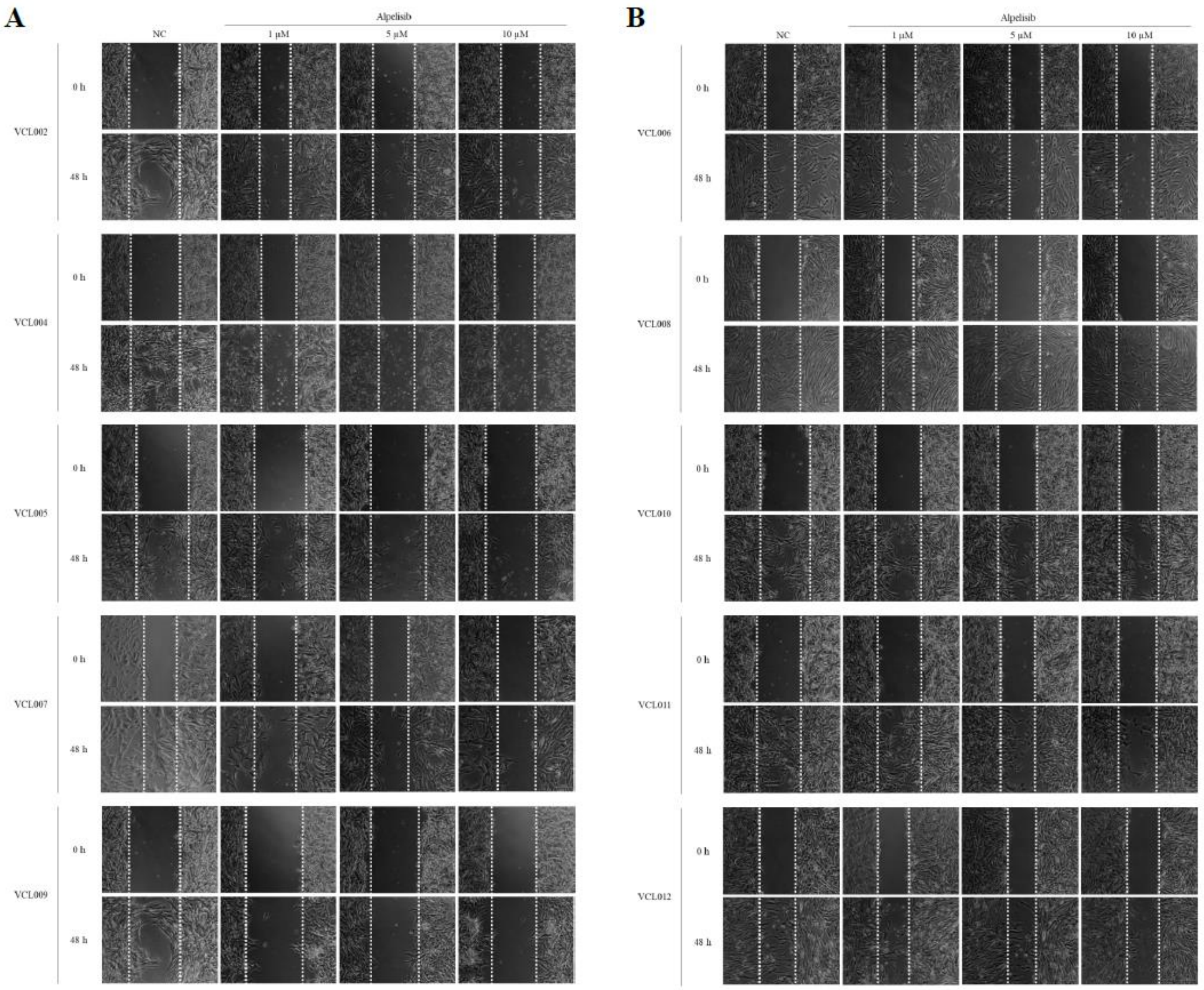

Supplementary Figure 2. The migration of VCLs were measured by the wound healing assay. After formation of scratch, the cells were treated with various concentrations of alpelisib (0, 1, 5, and 10 μM) for 48 h (4 ×, scale bar = 200 μm).
